# A Pivotal Role of Vitamin B9 in the Maintenance of Regulatory T Cells *In Vitro* and *In Vivo*

**DOI:** 10.1371/journal.pone.0032094

**Published:** 2012-02-20

**Authors:** Jun Kunisawa, Eri Hashimoto, Izumi Ishikawa, Hiroshi Kiyono

**Affiliations:** 1 Division of Mucosal Immunology, Department of Microbiology and Immunology, Institute of Medical Science, The University of Tokyo, Tokyo, Japan; 2 Department of Medical Genome Science, Graduate School of Frontier Science, The University of Tokyo, Chiba, Japan; 3 Core Research for Evolutional Science and Technology (CREST), Japan Science and Technology Agency, Tokyo, Japan; 4 Graduate School of Medicine, The University of Tokyo, Tokyo, Japan; Centre de Recherche Public de la Santé (CRP-Santé), Luxembourg

## Abstract

Dietary factors regulate immunological function, but the underlying mechanisms remain elusive. Here we show that vitamin B9 is a survival factor for regulatory T (Treg) cells expressing high levels of vitamin B9 receptor (folate receptor 4). In vitamin B9-reduced condition *in vitro*, Treg cells could be differentiated from naïve T cells but failed to survive. The impaired survival of Treg cells was associated with decreased expression of anti-apoptotic Bcl2 and independent of IL-2. *In vivo* depletion of dietary vitamin B9 resulted in the reduction of Treg cells in the small intestine, a site for the absorption of dietary vitamin B9. These findings provide a new link between diet and the immune system, which could maintain the immunological homeostasis in the intestine.

## Introduction

To achieve immunosurveillance and immunological homeostasis at the interface between the interior and exterior of the gastrointestinal tract, the intestinal immune system tightly balances states of immune activation and quiescence [1]. Thus, gastrointestinal tissues contain numerous kinds of T cells, such as Th1, Th2, Th17, forkhead box P3 (Foxp3)^+^ regulatory T (Treg) cells, IL-10–producing Foxp3^−^ T regulatory type 1 cells, and T cells expressing γδ T cell receptor, which together create the appropriate immunological environment.

Th17 and Treg cells are observed most frequently in the intestine, and their preferential differentiation is achieved by a unique cytokine environment created by transforming growth factor β (TGF-β), IL-6, and IL-23 [2]. In addition to these host-derived factors, the development and function of the immune system are influenced by crosstalk with environmental factors [3]. For example, stimulation by segmented filamentous bacteria results in the preferential induction of Th17 cells, whereas colonic Treg cells are induced by crosstalk between epithelial cells and Clostridium clusters IV and XIVa [4], [5], [6].

Nutritional molecules are also considered to be essential environmental factors for the development, maintenance, and regulation of gut immune responses. Thus, deficient or inappropriate nutritional intake increases the risk of infectious, allergic, and inflammatory diseases [7], [8]. Among various dietary factors, vitamins are important participants in the regulation of immune responses. For example, vitamin A is converted into retinoic acid (RA) by gut-associated dendritic cells; RA induces the expression of gut-homing molecules (e.g., α4β7 integrin and CCR9) on activated T and B cells [9], [10] and promotes the preferential differentiation of Treg cells and the simultaneous inhibition of Th17 cells [11], [12], [13], [14]. Vitamin B6 is required for the metabolic pathway of sphingosine 1-phosphate, a lipid mediator that regulates cell trafficking [15]; disruption of vitamin B6 function results in aberrant T-cell differentiation and cell trafficking in both systemic and intestinal compartments [16], [17], [18].

Vitamin B9 (also known as folate and folic acid) is a water-soluble vitamin derived from both diet and commensal bacteria [19]. Vitamin B9 is essential for the synthesis, replication, and repair of nucleotides for DNA and RNA and is thus required for cell proliferation and survival [20]. Methotrexate (MTX) acts as a vitamin B9 antagonist and blocks vitamin B9–mediated nucleotide synthesis, making MTX useful as an anti-tumor [21] and anti-rheumatoid arthritis agent [22]. Vitamin B9 deficiency also reduces the proliferative responses of lymphocytes and natural killer cell activity [23], [24]. Additionally, the vitamin B9 receptor folate receptor 4 (FR4) is both a marker of Treg cells and is immunologically functional [25]; however, how it functions in the intestinal immune system is largely unknown. In this study, we examined the role of vitamin B9 in the regulation of Treg cell *in vitro* and *in vivo*.

## Materials and Methods

### Mice and experimental treatment

Female Balb/c mice (7–9 wk of age) were purchased from Japan Clea (Tokyo, Japan). Vitamin B9–deficient and control diets composed of chemically defined materials (Oriental Yeast, Tokyo, Japan) were used within 3 months. All animals were maintained in the experimental animal facility at the University of Tokyo, and the experiments were approved by the Animal Care and Use Committee of the University of Tokyo and conducted in accordance with their guidelines (Approval #20–28).

### Lymphocyte isolation

Lymphocytes were isolated from the lamina propria (LP), as previously described [18], [26]. Briefly, lymphocytes were isolated from dissected PPs by enzymatic dissociation using collagenase (Wako, Osaka, Japan). To isolate lymphocytes from the LP of jejunum/duodenum, PPs were removed and the remaining intestinal tissue was cut into 2-cm pieces and stirred in RPMI 1640 medium containing 1 mM EDTA and 2% fetal calf serum (FCS). The tissue pieces were then stirred in 0.5 (for small intestine) or 1.0 (for large intestine) mg/mL collagenase, and the dissociated cells were subjected to centrifugation through a discontinuous Percoll gradient. Lymphocytes were isolated at the interface between the 40% and 75% Percoll layers.

### Flow cytometry and cell sorting

Flow cytometry and cell sorting were performed as previously described [18], [26]. Cells were pre-incubated with anti-CD16/32 antibodies and then stained with fluorescent antibodies specific for CD4, ICOS, and GITR (BD Biosciences, San Jose, CA) and FR4 (Biolegend). A Via-probe solution (BD Biosciences) was used to discriminate between dead and living cells. Intracellular staining of Foxp3 (eBioscience, San Diego, CA), phosphorylated STAT5, Ki67 and Bcl2 (BD Biosciences) was performed in accordance with the manufacturers' instructions. Flow cytometry and cell sorting were carried out using the FACSCantoII and FACSAria systems (BD Biosciences), respectively.

### Vitamin B9 measurement

To measure vitamin B9 concentrations, intestinal washes were collected by washing 12 cm of jejunum/duodenum or whole colon with 1 mL of PBS. The vitamin B9 concentration in intestinal washes and serum was measured with a RIDASCREEN enzyme immunoassay kit (R-Biopharm AG, Darmstadt, Germany) in accordance with the manufacturer's instructions. To measure the amounts of intracellular vitamin B9, 5×10^6^ purified cells were washed twice with PBS, and a cell lysate was obtained by homogenizing cells in PBS containing 0.01% NP-40. After cell debris was removed by centrifugation, vitamin B9 amounts in the supernatant were measured with a RIDASCREEN enzyme immunoassay kit.

### *In vitro* culture

For the induction of Treg cells from naïve T cells, CD62L^hi^CD4^+^ naïve T cells (10^5^ cells/well) were cultured for 4 days with 5 µg/mL of immobilized anti-CD3 antibody and 1 µg/mL of an anti-CD28 antibody (BD Biosciences) plus 2 ng/mL of human TGF-β (PeproTech, Rocky Hill, NJ) in vitamin B9–null or normal RPMI 1640 medium containing 10% FCS. To examine the maintenance of differentiated Treg cells, purified CD25^+^CD4^+^ T cells (10^5^ cells/well) were cultured for 4 days with 5 µg/mL of immobilized anti-CD3 antibody with or without 1000 units/mL of IL-2 (Peprotech) in vitamin B9–null or normal RPMI 1640 medium containing 10% FCS in the presence or absence of 100 nM MTX.

### Statistics

Results were compared with the Student's *t*-test by using GraphPad Prism (GraphPad Software, San Diego, CA). Statistical significance was established at *P*<0.05.

## Results

### Vitamin B9 is required for the survival of Foxp3^+^ Treg cells

**Figure 1:**
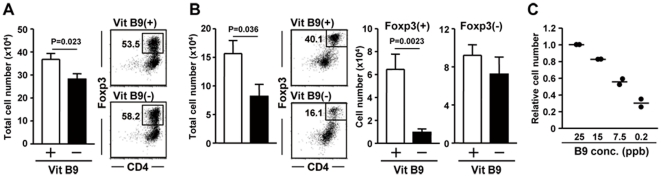
Requirement of vitamin B9 for the maintenance of Treg cells. (A) Purified naïve CD4^+^ T cells were stimulated with anti-CD3 and anti-CD28 antibodies plus TGF-β in the presence of normal [Vit B9(+)] or reduced [Vit B9(−)] amounts of vitamin B9. After 4 days, total cell numbers were calculated, and the differentiation into Foxp3^+^ Treg cells was examined by flow cytometry. Data are means ± SEM (n = 4). (B) CD25^+^CD4^+^ T cells were cultured with anti-CD3 antibodies in Cont or B9(−) medium. The frequencies of Foxp3^+^ and Foxp3^−^CD4^+^ T cells (B) were determined by flow cytometry. Cell numbers were calculated using the total cell number and flow cytometric data. Data are means ± SEM (n = 6). (C) Experiments similar to that shown in (B) were performed with different concentrations of vitamin B9. The relative cell number of Foxp3^+^ Treg cells is expressed as a ratio to the cell number in control medium. The values and means are indicated with dots and lines, respectively. Similar results were obtained from 2 independent experiments.

Foxp3^+^ Treg cells express high levels of FR4, which is essential for their maintenance [25]. We therefore examined whether vitamin B9 is required for the differentiation of Treg cells from naïve T cells, the survival of differentiated Treg cells, or both. To address this, we initially performed an *in vitro* T-cell differentiation assay. Purified naïve CD4^+^ T cells were stimulated with anti-CD3 and anti-CD28 antibodies plus TGF-β in complete or vitamin B9–reduced medium. Although a small amount of vitamin B9 is supplied from fetal calf serum (FCS) even in vitamin B9–null medium (0.2 ppb, compared with 25 ppb in normal medium), the total cell number was decreased in the condition with reduced vitamin B9 compared to the control; however, Foxp3^+^ Treg cells were generated at a normal frequency (Fig. 1A).

To investigate the effects of vitamin B9 on differentiated Treg cells, we cultured CD25^+^ Treg cells with anti-CD3 antibodies. The total cell number was significantly lower in the vitamin B9–reduced condition than in the control condition (Fig. 1B). The reduction in cell number occurred predominantly among the Foxp3^+^CD4^+^ Treg cells (Fig. 1B). The reduction of FR4^hi^Foxp3^+^ T cells was dependent on the dose of vitamin B9 (Fig. 1C).

**Figure 2:**
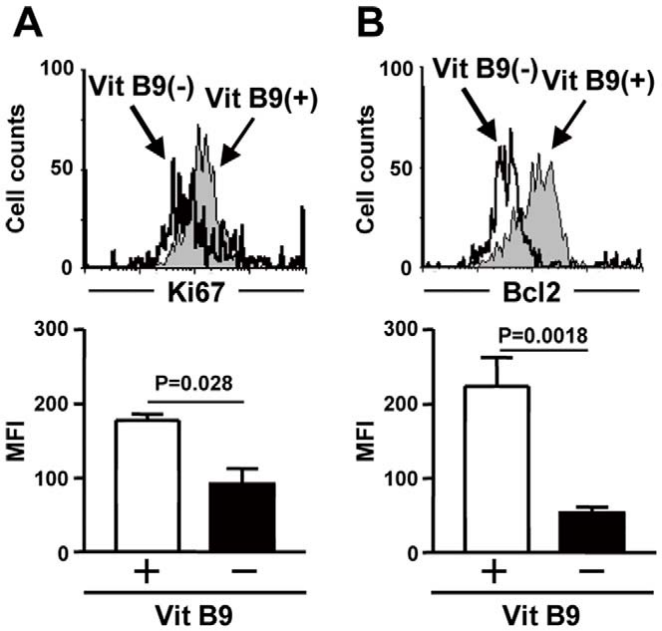
Vitamin B9 is essential for the survival of Treg cells. CD25^+^CD4^+^ T cells were cultured with anti-CD3 antibodies in Vit B9(+) or Vit B9(−) medium. The expression of Ki67 (A) and Bcl2 (B) in Foxp3^+^CD4^+^ T cells were determined by flow cytometry (top panels) and graphs show the means fluorescent intensity (MFI; bottom panels). Data are means ± SD (n = 3). Data are representative of 4 independent experiments.

We then measured the expression of Ki67 and anti-apoptotic Bcl-2 to investigate whether decreased number of Foxp3^+^CD4^+^ Treg cells in vitamin B9–reduced medium was due to the defects of cell proliferation, survival, or both. We found that both Ki67 and Bcl2 were decreased in Foxp3^+^CD4^+^ Treg cells cultured in vitamin B9 vitamin B9–reduced medium, but magnitude of Bcl2 reduction was higher than Ki67 reduction (Fig. 2A and B). These findings suggest that vitamin B9 is preferentially but not exclusively required for the survival of Treg cells *in vitro*.

### Vitamin B9 carrier-mediated pathway is not specifically involved in the survival of Treg cells

**Figure 3:**
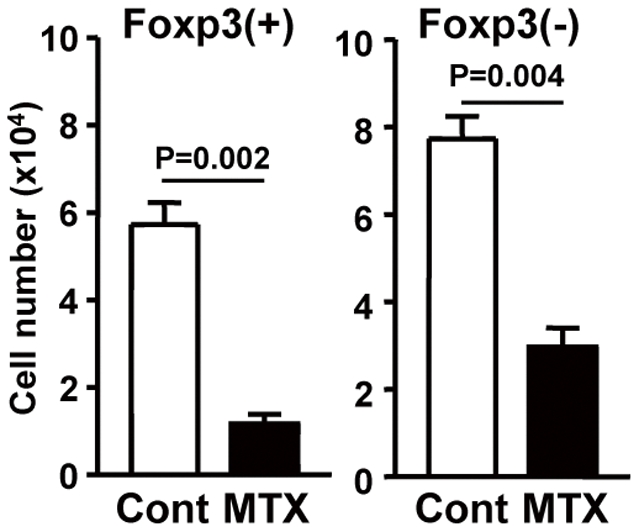
Vitamin B9 carrier-mediated pathway is not specific pathway in the maintenance of T cell survival. CD25^+^ CD4^+^ T cells were cultured with an anti-CD3 antibody in complete medium containing 100 nM methotrexate (MTX), and the frequency and absolute cell numbers of Foxp3^+^ and Foxp3^−^ CD4^+^ T cells were determined. Data are means ± SEM (n = 4). Data are representative of two independent experiments.

Because vitamin B9 is highly hydrophilic, mammalian cells must actively mediate the entry of vitamin B9 into cells by carrier- or receptor-mediated pathways [27]. Carriers include the proton-coupled folate transporter and the reduced folate carrier [27]. To examine whether a carrier-mediated pathway is involved in maintaining Treg cells, we employed MTX, an antagonist of vitamin B9 that is transported mainly via the reduced folate carrier and rarely via folate receptors [28], [29]. MTX treatment reduced the numbers of both Treg and non-Treg cells (Fig. 3), suggesting that the carrier-mediated pathway does not specifically maintain Treg cells.

### Vitamin B9 is an IL-2–independent survival factor for Treg cells

**Figure 4:**
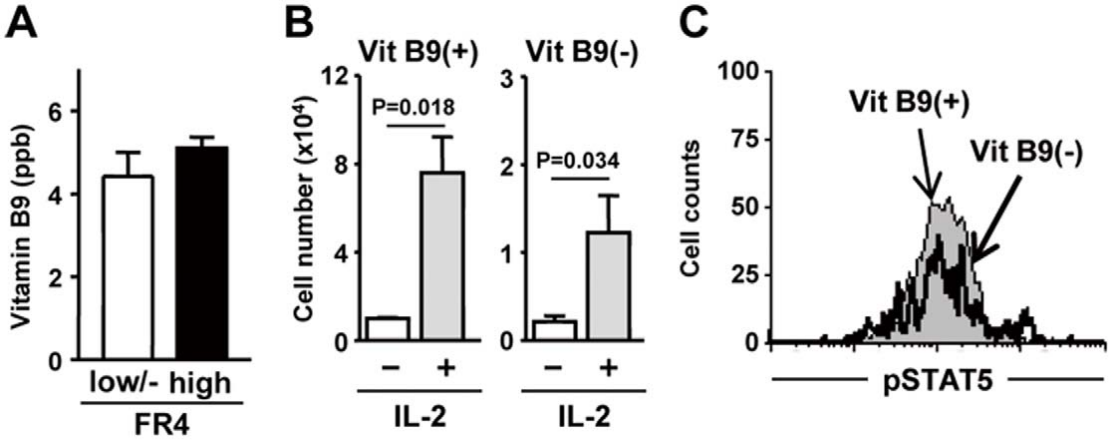
Vitamin B9 is IL-2-independent survival factor for Treg cells. (A) The amounts of intracellular vitamin B9 were measured using purified CD4^+^FR4^hi^ Treg or CD4^+^FR4^low/−^ non-Treg cells. Data are means ± SEM (n = 4). (B, C) Experiments similar to those shown in Fig. 1B were performed in the presence of anti-CD3 antibody stimulation with or without IL-2 stimulation. Cell number of Foxp3^+^CD4^+^ T cells (B) and the expression of phosphorylated STAT5 (pSTAT5) in Foxp3^+^CD4^+^ T cells (C) were determined. Data in (B) are means ± SEM (n = 6). Similar results were obtained from 3 separate experiments.

Treg cells could vigorously proliferate in some circumstances (e.g., antigen-specific activation through their highly sensitive TCR signaling [30] and IL-2-mediated activation [31]), which led to a hypothesis that Treg cells simply require large amounts of vitamin B9 as a source of nucleotides, and thus Treg cells might express FR4 as an additional means of acquiring vitamin B9. If so, FR4^hi^ Treg cells should contain a larger amount of vitamin B9 in the intracellular compartments; however, the amount of intracellular vitamin B9 was equivalent between FR4^hi^ Treg and FR4^low/−^ non-Treg cells (Fig. 4A). Thus, FR4 might have an additional specific function for the survival of Treg cells.

IL-2 stimulation enhance the survival of Treg cells [31], [32], [33]. The FR4-mediated vitamin B9 signal might undergo crosstalk with IL-2-mediated signaling to maintain the survival of FR4^hi^Foxp3^+^ Treg cells. To test this, Treg cells were cultured with an anti-CD3 antibody together with IL-2. Although the absolute cell numbers were low in the reduced vitamin B9 condition, the magnitude of the IL-2-mediated enhancement of Treg cell growth was similar in the control and vitamin B9-reduced conditions (Fig. 4B). Consistent with this finding, comparable expression of phosphorylated STAT5 was noted in the control and vitamin B9-reduced conditions (Fig. 4C).

### Dietary vitamin B9 maintains Foxp3^+^ Treg cells in the small intestine

**Figure 5:**
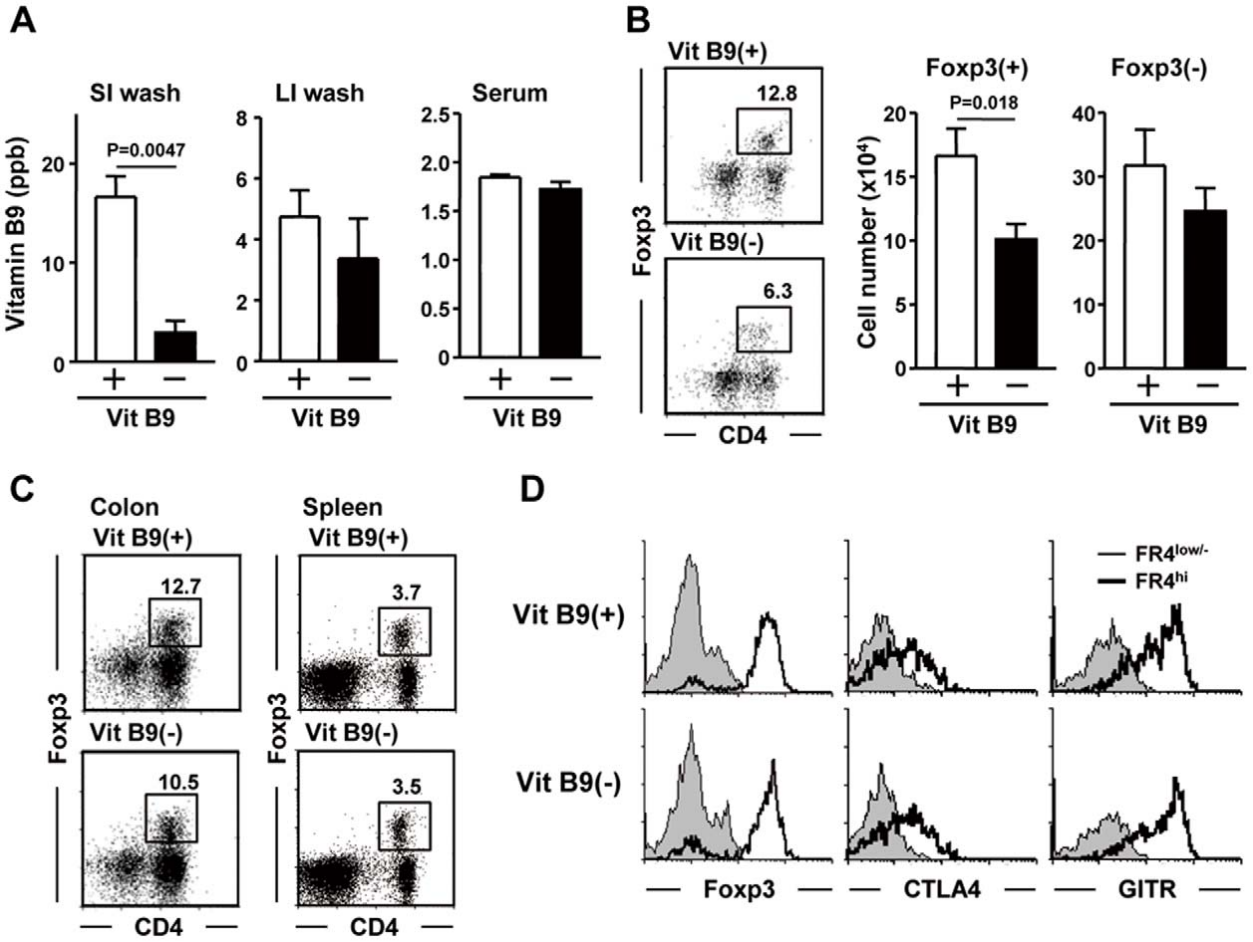
Depletion of dietary vitamin B9 selectively reduces Treg cells in the small intestine. Mice were maintained on a control [Vit B9(+)] or vitamin B9-depleted [Vit B9(−)] diet for 8 wk. (A) Vitamin B9 concentrations were measured in intestinal washes of the small intestine (SI), large intestine (LI), and serum. The data are mean ± SEM (n = 6). (B, C) The frequency and cell numbers of Foxp3^+^ and Foxp3^−^ CD4^+^ T cells in the small intestine (B), colon, and spleen (C) were calculated using the total cell number and flow cytometric data (mean ± SEM, n = 6). (D) Flow cytometric analysis was performed to determine the expression levels of Foxp3, CTLA4, and GITR on the surface of FR4^low/−^ (thin line) and FR4^hi^ (thick line) CD4^+^ T cells in the LP. Similar results were obtained from 3 separate experiments.

To examine whether vitamin B9 affects Treg cells *in vivo*, we maintained mice on a vitamin B9–depleted diet for 8 wk. Mice maintained with vitamin B9(−) diet showed less vitamin B9 in the small-intestinal wash than controls (Fig. 5A). In contrast, the amounts of vitamin B9 in the large-intestinal wash and serum were not different in those mice (Fig. 5A), presumably due to vitamin B9 production from commensal bacteria [19].

We then focused on Treg cells in the mice maintained with vitamin B9(−) diet. Consistent with our *in vitro* data, the small intestines of mice maintained with vitamin B9(−) diet had fewer Foxp3^+^ Treg cells than those of control mice (p = 0.018), and there was no statistical difference (p = 0.3022) in the number of Foxp3^−^CD4^+^ non-Treg cells (Fig. 5B). The number of Treg and non-Treg cells was not significantly changed in the colon and spleen of mice maintained with vitamin B9(−) diet (Fig. 5C), which could be explained by the similar concentration of vitamin B9 in the large-intestinal washes and sera of both groups of mice. We also found that Foxp3 and the inhibitory molecules CTLA4 and GITR, which are specifically expressed on Treg cells, were comparable between those mice (Fig. 5D).

## Discussion

We have shown that vitamin B9 is crucial for the maintenance of Treg cells. Intriguingly, vitamin B9 was required for the survival of differentiated Treg cells, but was not necessary for the differentiation of naïve T cells into Treg cells. This selective effect of vitamin B9 on Treg cells is opposite to the effect of RA, a vitamin A metabolite, which enhances the differentiation of naïve T cells into Treg cells [11], [12], [13], [14]. RA also induces the expression of gut-homing molecules (e.g., α4β7 integrin and CCR9) on B and T cells activated by gut dendritic cells [9], [10]. Because CCR9 was expressed normally on Treg cells in the LP of mice maintained with vitamin B9(−) diet (data not shown), the deficiency of dietary vitamin B9 did not affect the RA-mediated expression of gut-homing molecules and, predictably, the induction of Treg cells in the small intestine.

Treatment with the vitamin B9 antagonist MTX affected survival of both Treg cells and non-Treg cells, suggesting that the carrier-mediated pathway maintains sufficient amounts of intracellular vitamin B9 for cell survival regardless of the T-cell subset. The indiscriminate effects of MTX could be explained by the ubiquitous expression of the folate carrier [29], [34]. As the mechanism of FR4-mediated Treg-cell maintenance, we considered initially that the proliferative activity of Treg cells could require large amounts of vitamin B9 as a source of nucleotides for DNA and RNA. However, the amounts of intracellular vitamin B9 were identical between Treg and non-Treg cells, implying that FR4 specifically recognizes extracellular vitamin B9 for the maintenance of Treg cell survival, consistent with a report that FR4 expressed on Treg cells contributes to their immune function and survival [25]. Additionally, the specific biological functions of vitamin B9 receptors (FR1, FR2, and FR4) have been predicted on the basis of their ∼70% amino acid sequence identity, but the expression of each receptor is rigidly restricted, with narrow tissue and cell specificity [35], [36]. Because FR1, FR2, and FR4 are glycosyl phosphatidylinositol–anchored proteins [37], adapter molecules may assist FR4 in the maintenance of Treg cell survival. We found that vitamin B9/FR4 was not associated with IL-2–mediated signaling in Treg cells. We will continue to study how FR4-mediated vitamin B9 regulates the survival of Treg cells.

Mammals must obtain vitamin B9 from the diet or from commensal bacteria. The absorption of vitamin B9 from the diet occurs mainly in the small intestine, whereas the uptake of microbial vitamin B9 predominantly occurs in the colon [38]. This explains why depletion of dietary vitamin B9 specifically decreased Treg cells in the small intestine, but not in the colon. It has been proposed that bacterial vitamin B9 absorbed in the colon affects the vitamin B9 status of the host [39], [40], which may explain the lack of changes in vitamin B9 in the serum and splenic Treg cells in mice maintained with vitamin B9(−) diet. *Bifidobacterium*, one of the most important genera of commensal bacteria to be used as a probiotic, is well-studied as a vitamin B9 producer [41], and colonic Treg cells are specifically induced by immunological crosstalk with commensal bacteria, especially *Clostridium* clusters IV and XIVa [5]. Although whether *Clostridium* clusters IV and XIVa produce vitamin B9 remains unclear, our current findings suggest that vitamin B9 is an essential survival factor for Treg cells and, in vivo situation, diet vitamin B9 establishes an immunological network in the maintenance of Treg cells specifically in the small intestine.
